# Surgeons’ physical workload in open surgery versus robot-assisted surgery and nonsurgical tasks

**DOI:** 10.1007/s00464-022-09256-0

**Published:** 2022-05-19

**Authors:** Xuelong Fan, Mikael Forsman, Liyun Yang, Carl M. Lind, Magnus Kjellman

**Affiliations:** 1 IMM Institute of Environmental Medicine, Karolinska Institutet, 171 77 Stockholm, Sweden; 2 Division of Ergonomics, School of Engineering Sciences in Chemistry, Biotechnology and Health, KTH Royal Institute of Technology, Hälsovägen 11C, 14157 Huddinge, Sweden; 3 Centre for Occupational and Environmental Medicine, Stockholm County Council, 113 65 Stockholm, Sweden; 4 Department of Molecular Medicine and Surgery, Department of Environmental Medicine, Karolinska Institutet, 171 77 Stockholm, Sweden

**Keywords:** Surgical ergonomics, Inclinometry, Muscle activity, Rationalization, Task-based analysis, Musculoskeletal disorders

## Abstract

**Background:**

Musculoskeletal disorders (MSDs) are common among surgeons, and its prevalence varies among surgical modalities. There are conflicting results concerning the correlation between adverse work exposures and MSD prevalence in different surgical modalities. The progress of rationalization in health care may lead to job intensification for surgeons, but the literature is scarce regarding to what extent such intensification influences the physical workload in surgery. The objectives of this study were to quantify the physical workload in open surgery and compare it to that in (1) nonsurgical tasks and (2) two surgeon roles in robot-assisted surgery (RAS).

**Methods:**

The physical workload of 22 surgeons (12 performing open surgery and 10 RAS) was measured during surgical workdays, which includes trapezius muscle activity from electromyography, and posture and movement of the head, upper arms and trunk from inertial measurement units. The physical workload of surgeons in open surgery was compared to that in nonsurgical tasks, and to the chief and assistant surgeons in RAS, and to the corresponding proposed action levels. Mixed-effects models were used to analyze the differences.

**Results:**

Open surgery constituted more than half of a surgical workday. It was associated with more awkward postures of the head and trunk than nonsurgical tasks. It was also associated with higher trapezius muscle activity levels, less muscle rest time and a higher proportion of sustained low muscle activity than nonsurgical tasks and the two roles in RAS. The head inclination and trapezius activity in open surgery exceeded the proposed action levels.

**Conclusions:**

The physical workload of surgeons in open surgery, which exceeded the proposed action levels, was higher than that in RAS and that in nonsurgical tasks. Demands of increased operation time may result in higher physical workload for open surgeons, which poses an increased risk of MSDs. Risk-reducing measures are, therefore, needed.

Musculoskeletal disorders (MSDs) have been a common health issue among surgeons for decades [1]. Compared to most occupations, surgeons have a relatively high prevalence of MSDs, and this trend is persistent across many countries [2–9]. The neck, shoulder, upper back and lower back are the most common body sites of pain and discomfort among surgeons [1]. The distribution of the effected body sites differs by surgical specialty [10], e.g., gynecology, gastrointestinal surgery and urological surgery, and by surgical modality [1, 10, 11], e.g., open surgery, laparoscopic surgery, endoscopic surgery and robot-assisted surgery (RAS). For example, surgeons performing minimally invasive procedures, such as laparoscopic and robotic surgery, have reported a higher prevalence of pain in the neck and the shoulders than surgeons performing open surgeries [1].

Awkward postures and high muscle activity levels have been associated with MSDs [12, 13]. A few studies have observed a decrease in exposure to awkward neck and shoulder postures (i.e., postural loads) among surgeons when moving from open surgeries to minimally invasive surgeries [14, 15]. Among minimally invasive surgeries, laparoscopic surgery was reported to be associated with lower muscle activity levels in the shoulders than RAS [16]. Lower levels of muscular activation in neck and shoulder muscles were also observed among surgeons who performed laparoscopic surgeries than among those performing open surgeries [17]. The lower postural loads and muscle activities among surgeons performing minimally invasive surgeries, especially laparoscopic surgery, should in theory lead to a lower prevalence of MSDs. Despite this, a higher prevalence of neck and shoulder pain has been reported among surgeons performing minimally invasive surgery than among surgeons performing open surgery [1]. Additionally, shoulder pain has been reported to be more prevalent when performing laparoscopic surgery than when performing RAS [10].

These contradictory results may be explained by differences in the proportion of time in which there is sustained muscle activation, which can cause prolonged activation of single (muscle) motor units, so-called Cinderella units [18, 19]. This sustained muscle activation can be quantified by a measurement parameter that is called sustained low-level muscle activity (SULMA) [20], which have been associated with an increased prevalence of work-related MSDs [21]. For surgeons, differences in patterns of sustained muscle activation have been observed between those who performed laparoscopic surgeries and those who performed RAS [16]. However, no studies have, to the best of our knowledge, presented SULMA among surgeons who perform open surgery or among other medical personnel. Analyzing such data could potentially provide important information to clarify the current contradictory findings.

Additionally, following the progress of rationalization in the industry and public service sectors, the impacts of increasing production efficiency or the proportion of value-adding work (VAW) on healthcare workers were widely studied in various occupations [22]. Among those, the efficiency of operating rooms in hospitals has been highlighted for its direct linkage to the financial statuses of hospitals [23], for its complex relation with surgeon behaviors [24], and for its relationship to team dynamics in the operating room [23]. However, the heavy focus on increasing efficiency is associated with work intensification [25], which has been associated with increased risks of MSDs among healthcare workers [26]. Few studies have addressed the extent to which this intensification affects surgeons.

To scrutinize the potential effect of work intensification, a task-based analysis can be used to quantify single elements of the job to predict their individual contribution in a work intensification context. This approach with workload assessment offers an informative map of how the workload is distributed within an occupation. The application of such a method in medical fields can help to address work intensification issues among medical workers, e.g., dentists and dental hygienists [27, 28]. However, recent studies measuring task-based exposure during surgery are lacking.

From open surgery, to laparoscopic surgery and RAS, the spatial separation of patients and surgeons are introduced and enlarged by the development of surgical tools. This evolvement allows a decoupling of traditional tasks of surgeons, such as commanding and executing. To better understand the effects and impacts of this transition on surgeons, it is helpful to examine the differences between the start (open surgery) and the end (RAS) of this transition. Since the chief surgeons in RAS only directly interact with the console rather the patient, the physical workload of the surgeons in RAS is more affected by the design of the console, such as the model of the visual display unit [29], rather than the specialty of the surgery. Therefore, when comparing surgeons’ physical workload in cases of RAS to that in open surgery, it is less important to match the specialty of surgeries. As a result, in this study neck surgery was used as a representative of open surgery, and surgery in urology was used for representing RAS. The aims of this study were, using proposed action levels as references, 1) to quantify and compare physical workload in terms of head, trunk and arm kinematics, and trapezius muscle activity among open surgeons using a task-based analysis and 2) to compare trapezius muscle activity among surgeons in open surgery and those in RAS.

## Materials and methods

### Participants

Twenty-two surgeons, 12 surgeons specializing in open neck surgery (hereafter, referred to as open neck surgeons) and 10 urologists, participated in this study. More information on the urology surgeons is published elsewhere [30]. Open neck surgeons were recruited from the endocrine surgery units of two academic hospitals in Sweden from 2015 to 2019. Written informed consent was obtained from all participants. The demographic information of the participants, including age, sex, height, weight, glove size, dominant hand and surgical experience was collected. The study protocol was approved by the Ethical Review Board in Stockholm (dnr. 2015/167-32, extended from dnr: 2014/1120-31).

### Measurement protocol

The study consisted of two parts: (I) task comparisons and (II) modality comparisons.

For the task comparisons (part I), the open neck surgeons were followed by a researcher for an entire surgical workday. A surgical workday is defined as a workday when all work tasks are surgery oriented. This is in contrast with other administrative workdays or teaching workdays that involve research work, meetings and teaching, which were not included in this study. The surgeons in this study typically had three surgical workdays in a typical work week. Within a surgical workday, the following six main tasks were identified: surgery, surgical preparation, desk work, ward rounds, miscellaneous tasks (transportation between hospital parts, short breaks for bathroom visits, and other incidental events), and nonwork activities (e.g., breaks and meals). The first five identified tasks were further categorized as work tasks, contrasting nonwork activities (see Table 1). Among the work tasks, only surgery was defined as VAW; hence, all the other work tasks were considered non-VAW. Miscellaneous tasks were excluded from the task workload comparison. The researcher noted the type of tasks performed and the start and end times of those tasks.

**Table 1:** The six main identified categories of tasks, their description, and their short form used in the analysis (in parentheses)

Tasks	Definitions and descriptions
Work tasks (work)	All the tasks that occur in work areas
Value-adding work	
Surgery (surgery)	Operation time started from the first cut and ended when the surgeon removed the surgical gown
Non value-adding work	
Surgical preparation (preparation)	Preparation before surgery including checking patient conditions in the operating room, drawing marks and lines, washing hands, and putting on a surgical gown
Desk work (desk work)	Administrative or documentation time when the surgeon works on a computer before and after surgery
Ward rounds (rounds)	The time when surgeons check on operated patients in a ward after operation
Miscellaneous tasks (other)	Including transportation between sites, short breaks for bathroom visits, and other incidents that are outside the definitions of the above tasks but inside the surgical work area
Nonwork activities (nonwork)	
Breaks and meals	Including breakfast, coffee breaks, and lunch

For the modality comparisons (part II), both the surgeons specializing in open neck surgery and the urologists were followed by a researcher only during surgeries in the same way as that reported in a previous study [30]. The start and end times of each surgical case were noted.

### Comparisons of physical workload

The physical workload evaluated in this study include muscle activity, postures and movements. For the task comparison (part I), the muscle activity, working postures and movements of open neck surgeons were compared between surgical tasks and nonsurgical tasks and between work tasks and nonwork activities. For the modality comparison (part II), the muscle activity of the open neck surgeons was compared to those of the chief and assistant surgeons in urology performing RAS. All relevant measures of the physical workload were compared to the proposed action levels [31].

### Measurements of muscle activity

Muscle activity was recorded bilaterally by surface electromyography (sEMG) from the upper trapezius using self-adhesive bipolar electrodes with gel (Ag/AgCl electrodes, N-00-S/25, Ambu A/S, Copenhagen, Denmark). Each electrode pair had a center-center distance of 2 cm and was positioned 2 cm laterally to the midpoint from the C7 vertebra to the acromion process [32]. To increase the conductance, the skin was rubbed with an alcohol patch before the application of electrodes. The electrodes were connected to a data logger (Mobi8, from TMSi, Oldenzaal, The Netherlands) via actively shielded cables, and fixated with gel on the skin to prevent relocation of the electrodes during any movement [33]. The sEMG signals were sampled at 1024 Hz per channel with a 24-bit AD convertor and saved in a data logger (Mobi8, from TMSi, Oldenzaal, The Netherlands).

**Fig. 1:**
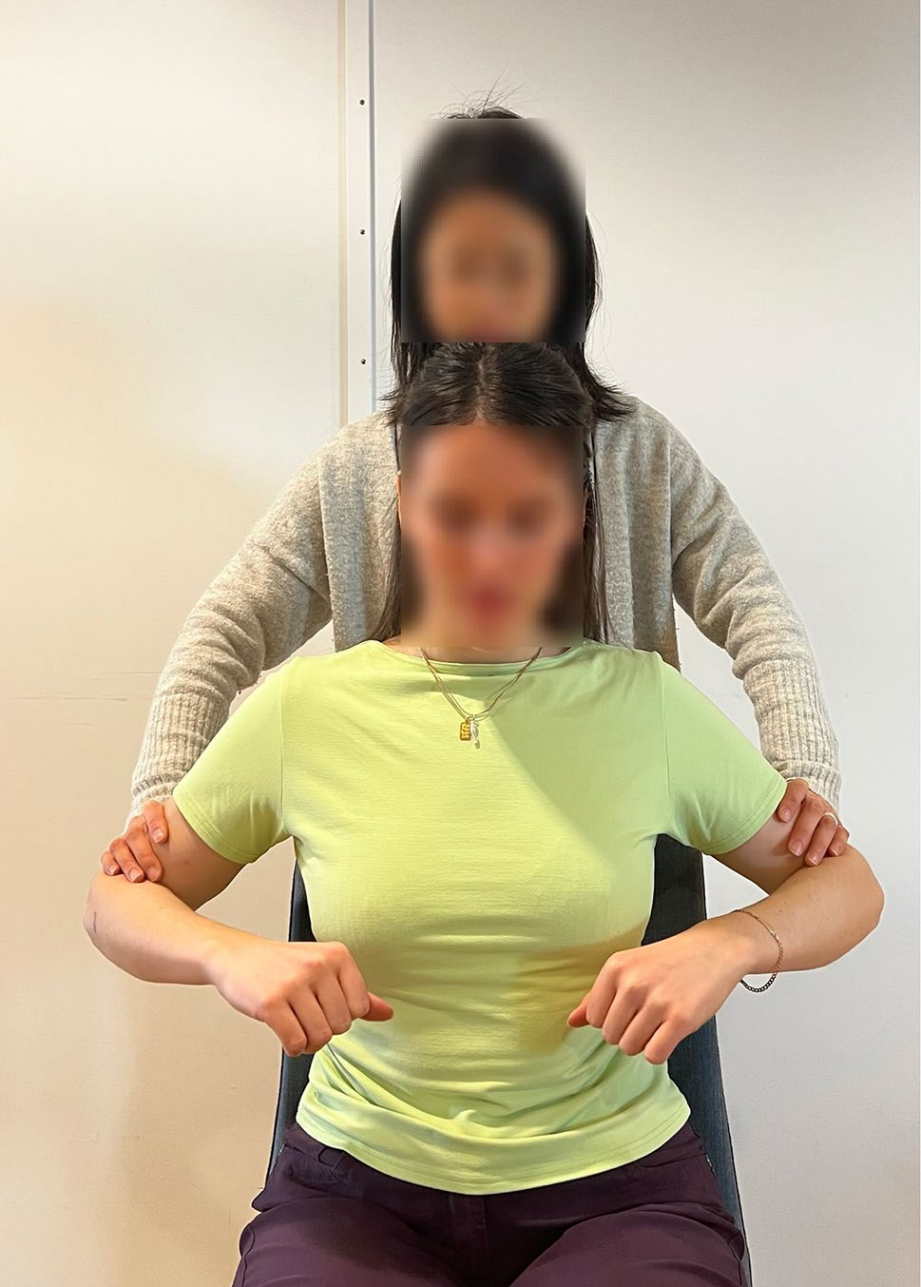
Maximal voluntary contraction (MVC) for normalization of EMG signals in trapezius

Maximal voluntary electrical activation (MVE) measurements were acquired from each participant for normalization. It was measured from three maximal voluntary contractions (MVCs) for each participant after the electrodes were mounted [32]. To perform the MVCs, the subjects were seated on a chair with both upper arms abducted to 45°; they then elevated the shoulder against external resistance on the upper arms applied by a researcher (see Fig. 1). The process was repeated three times. MVE was defined as the maximal value of the sEMG signals during the three MVCs.

### Measurements of posture and movement

Recording of working postures and movements was performed using an OPAL system (APDM, Inc., Portland, OR, USA), which included four inertial measurement units (IMUs). Only the accelerometer data were used in this analysis. The sampling rate was 64 Hz. The IMUs were positioned at the back of the head, on the sternum, and on both upper arms, approximately at the distal insertion of the deltoideus muscle [34].

The IMUs were positioned with double-sided adhesive tape and fixed with surgical tape. For calibration of the system, a series of reference postures were obtained, including the I-pose (standing straight with arms hanging downwards on the side) and the T-pose (standing straight with arms stretched out and held horizontally and laterally). The reference position for the upper arm (0° elevation) was obtained by having the subject lean toward the side (lateral trunk flexion) with relaxed arms hanging downward without touching the hip while holding a 2 kg dumbbell in the hand with the palm facing toward the body [35]. This procedure was performed bilaterally.

### Data processing

The EMG signals were processed with a digital bandpass filter (30–400 Hz), and the root mean square (RMS) values of the EMG signals were then calculated for each 1/8 s epoch [36]. Each individual MVE was chosen as the maximal RMS within the three MVCs [36]. Based on the RMS values, muscle activity was first calculated as the percentage of MVE (%MVE) [37]. After that, muscle activity was assessed via the level of muscle activity, muscle rest time and SULMA time. The levels of muscle activity were defined by an amplitude probability distribution function (APDF) as the 10^th^, 50^th^ and 90^th^ percentiles of the measured %MVE within a defined task [37]. The three percentiles are also often referred to as the static, median and peak levels of muscle activity [38, 39]. The muscle rest time was defined as the proportion of time when the muscle activity was below 0.5%MVE [36]. The SULMA time was defined as the proportion of time when the muscle activity was above 0.5%MVE for 8 consecutive minutes in a given measured period; a moving 1.6-s root mean square window was used to smooth the data before the calculation [20].

Postures were defined as the angles of a body part during a defined motion. For the head and trunk, the angle was the sagittal inclination angle of the head and the trunk, with positive values indicating ‘forward’ inclination; for the nondominant and dominant upper arms, the angle was defined as the elevation angle, that is, any inclination, of the arm from the reference zero-posture (see above). Movements of the head and the trunk were calculated as derivatives of the corresponding angles; movements of the upper arms were calculated as generalized velocity [40, 41]. The postures and movements of a body part were further summarized as the 10th, 50th and 90th percentiles of the angles within a task, the 50th percentile of the velocities within a task, and a neutral posture time (i.e., the proportion of time in a neutral posture as defined in Table 2) [42].

**Table 2:** Definition of a neutral posture including postural range and angular velocity for the head, trunk and upper arms

	Neutral posture (range)	Low velocity (°/s)
Head	0°–20°	< 5
Trunk	0°–20°	< 5
Upper arm	< 20°	< 5

### Statistical analysis

Linear mixed-effects (LME) models were used to compare differences between tasks and between modalities [15]. This approach was chosen to handle data that was unpaired and unbalanced data due to technical reasons and schedule variances and to maximize the usage of information. Based on the model used in previous studies [15, 43], two models were built:

1. For the task comparison (part I), the dependent variable was the measured workload, i.e., the muscle activity levels (the 10th, 50th and 90th percentile of %MVE) and muscle rest time of the trapezius of the dominant and nondominant sides, the postures of the head, trunk and upper arms (the 10th, 50th and 90th percentile of corresponding angles), and the movements of those body parts (the 50th percentile of corresponding angular velocities). The fixed effect was the measured task (surgery, preparation, desk work and rounds, or work and nonwork). Surgery was used as the reference. The participant was used as the random effect. Thus, the model was as follows:
  1 $${\mathrm{Workload}}_{ijm}= {\beta }_{0(m)}+ {\beta }_{1j(m)}{ \mathrm{Task}}_{j}+{b}_{i(m)}{\mathrm{Participant}}_{i}+ \varepsilon$$
  where ${\mathrm{Workload}}_{ijm}$ is the mth measured workload of the *i*th participant for the *j*th task, ${\beta }_{0(m)}$ is the intercept of the model for the *m*th measured workload, ${\beta }_{1j(m)}$ is the fixed effect of the mth measured workload for the *j*th task, ${Task}_{j}$ is the dummy variable for the *j*th task, ${b}_{i(m)}$ is the random effect of the *m*th measured workload for the *i*th participant, ${Participant}_{i}$ is the dummy variable for the ith participant, and $\varepsilon$ is the residual.
2. For the modality comparison (part II), the dependent variable was the measured workload, i.e., the muscle activity levels (the 10th, 50th and 90th percentile of %MVE) and muscle rest time of the trapezius of the dominant and nondominant sides. The fixed effect was a combination of the modality of the surgery and the surgeon’s role, i.e., open surgery, RAS (chief), and RAS (assistant). Open surgery was used as the reference. The participant was used as a random effect. The model was as follows:
  2 $${\mathrm{Workload}}_{ijm}= {\beta }_{0(m)}+ {\beta }_{1j(m)}{ \mathrm{Modality}}_{j}+{b}_{i(m)}{\mathrm{Participant}}_{i(m)}+ \varepsilon$$
  where ${\mathrm{Workload}}_{ijm}$ is the mth measured workload of the *i*th participant for the *j*th modality, ${\beta }_{0(m)}$ is the intercept of the model for the mth measured workload, ${\beta }_{1j(m)}$ is the fixed effect of the *m*th measured workload for the *j*th modality, ${Modality}_{j}$ is the dummy variable for the jth modality, ${b}_{i(m)}$ is the random effect of the mth measured workload for the ith participant, ${Participant}_{i}$ is the dummy variable for the *i*th participant, and $\varepsilon$ is the residual.

The normality of the residuals was examined via quantile–quantile plot, and the homogeneity of residuals was examined via scale-location plot. Since the assumptions of both normality and homogeneity were not fulfilled, a rankit transformation was performed on the data [44]. After rankit transformation, the homogeneity assumption was fulfilled, but the data were still nonnormally distributed. Since the LME model approach is robust to nonnormally distributed data [45], LME models were used. Since the variances calculated by maximum likelihood were similar to those calculated by restricted maximum likelihood, maximum likelihood was used [46]. Significant differences were identified when the fixed effect was significantly unequal to zero.

All statistical analyses were performed in MATLAB R2019b (The MathWorks, Inc., Natick, MA, USA). A significance level of 0.05 was used.

## Results

The distribution of the 22 participants is shown in Table 3. Two of the open neck surgeons were included in both the task comparison (part I) and the modality comparison (part II).

**Table 3:** The number of participants and measurements in each part of the study

Part I: Task comparison					
Task	Specialty	Subjects (*N*)		Measurements (*N*)	
		EMG	IMU	EMG	IMU
Work	Otorhinolaryngology	8	9	11	13
Surgery		8	9	11	13
Preparation		8	9	11	13
Desk work		8	9	11	13
Rounds		7	7	9	10
Nonwork		8	9	10	12

Part II: Modality comparison					
Surgical modality	Specialty	Subjects (*N*)		Surgical cases (*N*)	
Open	Otorhinolaryngology	5		25	
Robotic (chief)	Urology	6		11	
Robotic (assistant)		4		13	

The demographic information is presented in Table 4. All surgeons had at least 2 years of experience in surgery.

**Table 4:** The demographics of the participants by comparison groups

Characteristics	Task comparisons (including 9 surgeons)	Modality comparisons (of 15 surgeons)		
		Open surgery	Robotic surgery	
			Chief role	Assistant role
Male, *N* (%)	6 (67%)	3 (60%)	5 (83%)	3 (75%)
Age, mean (SD) years	48.4 (10.6)	46.6 (4.0)	47.7 (6.7)	40.3 (2.1)
Right-handed, *N* (%)^a^	8 (89%)	4 (80%)	6 (100%)	4 (100%)
Statue, mean (SD) cm	176.3 (9.9)	171.6 (6.1)	179.0 (7.4)	174.5 (13.3)
Weight, mean (SD) kg	77.0 (17.3)	68.2 (11.5)	80.8 (11.2)	79.5 (7.4)
BMI, mean (SD) kg/m^2^	24.5 (3.5)	23.0 (2.7)	25.2 (2.7)	26.3 (3.1)
Years employed, mean (SD) year	15.2 (9.6)	10.0 (5.6)	13.7 (8.7)	7.1 (4.9)

^a^Including 1 subject who was ambidextrous

### Part I: Task comparison

**Fig. 2:**
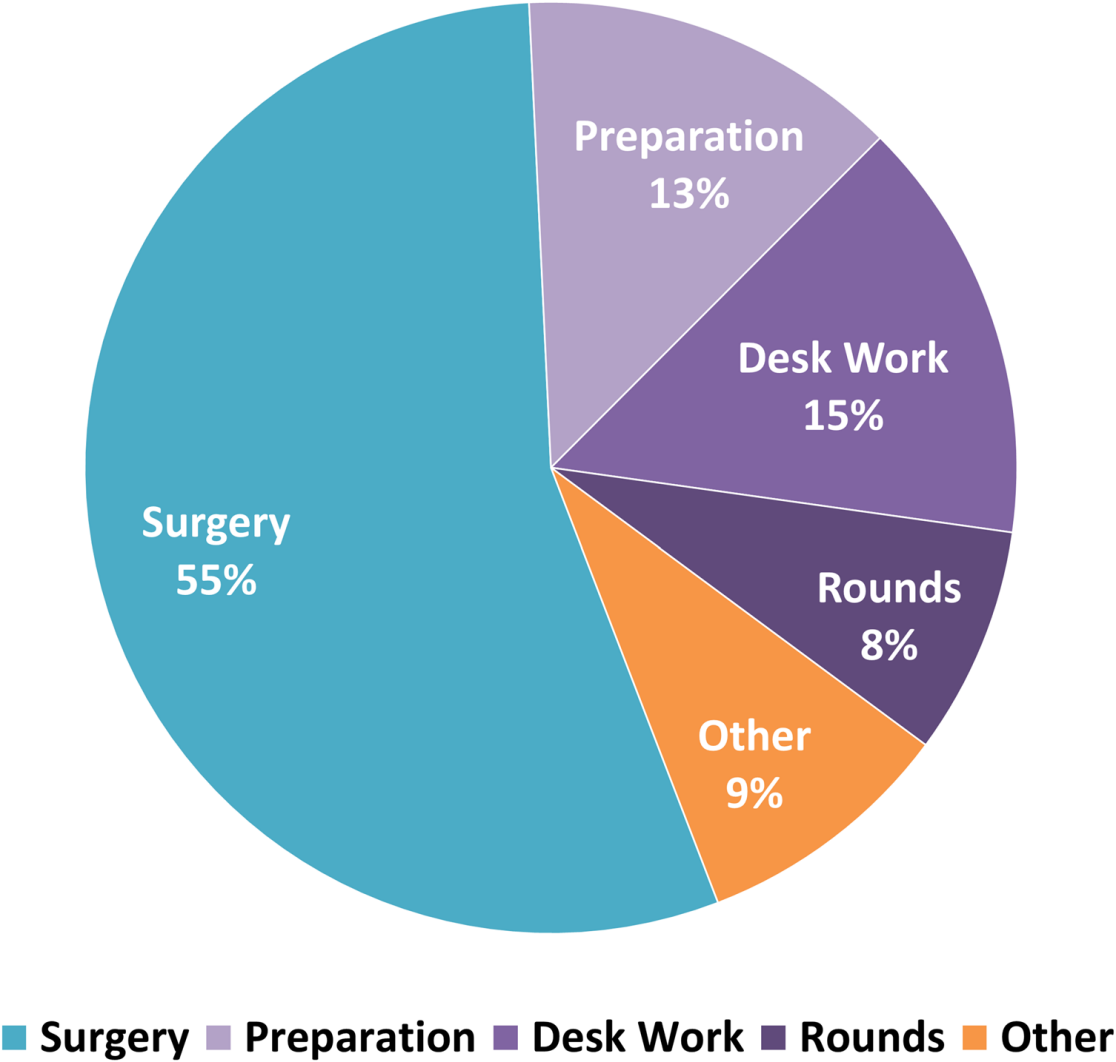
Distribution of the average duration of work tasks

Work tasks had an average duration of 277 ± 84 min (mean ± SD), which corresponded to 82% of the measurement period, while the average duration of the nonwork activities was 60 ± 29 min, which corresponded to 18% of the measurement period. Surgery constituted 55% (155 ± 76 min) of the total work time, followed by desk work at 15% (42 ± 25 min), surgical preparation at 13% (37 ± 21 min), rounds at 8% (22 ± 12 min), and other activities in the work area at 9% (25 ± 25 min) (Fig. 2).

**Fig. 3:**
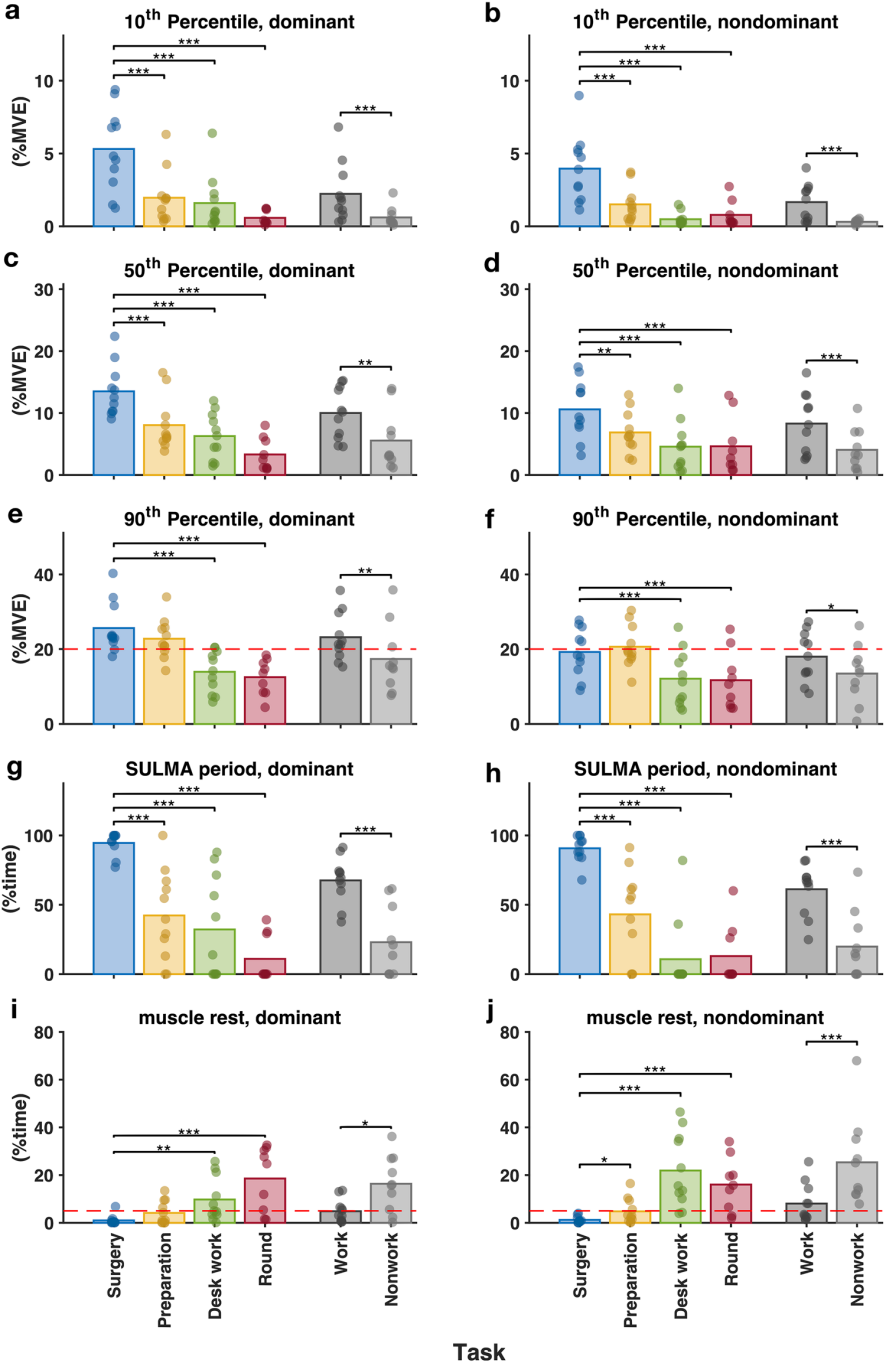
Comparisons of muscle activity in the upper trapezius between individual tasks and between work and nonwork activities. The bars represent the mean value of the group and the filled circles represent the individual value of the group. The red dashed line denotes the action level proposed by Arvidsson, Dahlqvist [30]. *p* values are denoted as * < 0.05; ** < 0.01; *** < 0.001 (Color figure online)

Figure 3 shows a comparison of the distribution of trapezius muscle activity between surgery and nonsurgical tasks and between work tasks and nonwork activities.

Surgery involved significantly higher trapezius muscle activity at the static and median levels on both the dominant and nondominant sides than all three other nonsurgical tasks (i.e., surgical preparation, desk work and rounds). The peak level of trapezius muscle activity during surgery was significantly higher bilaterally than that during desk work and rounds, and it was significantly higher than the proposed action level for the peak muscle activity (20%MVE) on the dominant side.

During surgery, the proportion of SULMA time of the trapezius was significantly higher than that during all the other three nonsurgical tasks, i.e., preparation, desk work and rounds, on both sides. On the nondominant side, the trapezius muscle rest time during surgery was significantly lower than that during all three nonsurgical tasks. On the dominant side, the trapezius muscle rest time during surgery was significantly lower than that during desk work and rounds but not that during preparation. Nevertheless, the muscle rest time of the trapezius during surgery was significantly lower than the proposed action level for the muscle rest time (5%) on both sides.

Compared to nonwork activities, work tasks had significantly higher static, median and peak trapezius muscle activity levels. The proportion of SULMA time of the trapezius was significantly higher during work tasks than during nonwork activities on both sides. The muscle rest time of the trapezius on both sides was significantly lower during work than during nonwork activities.

**Fig. 4:**
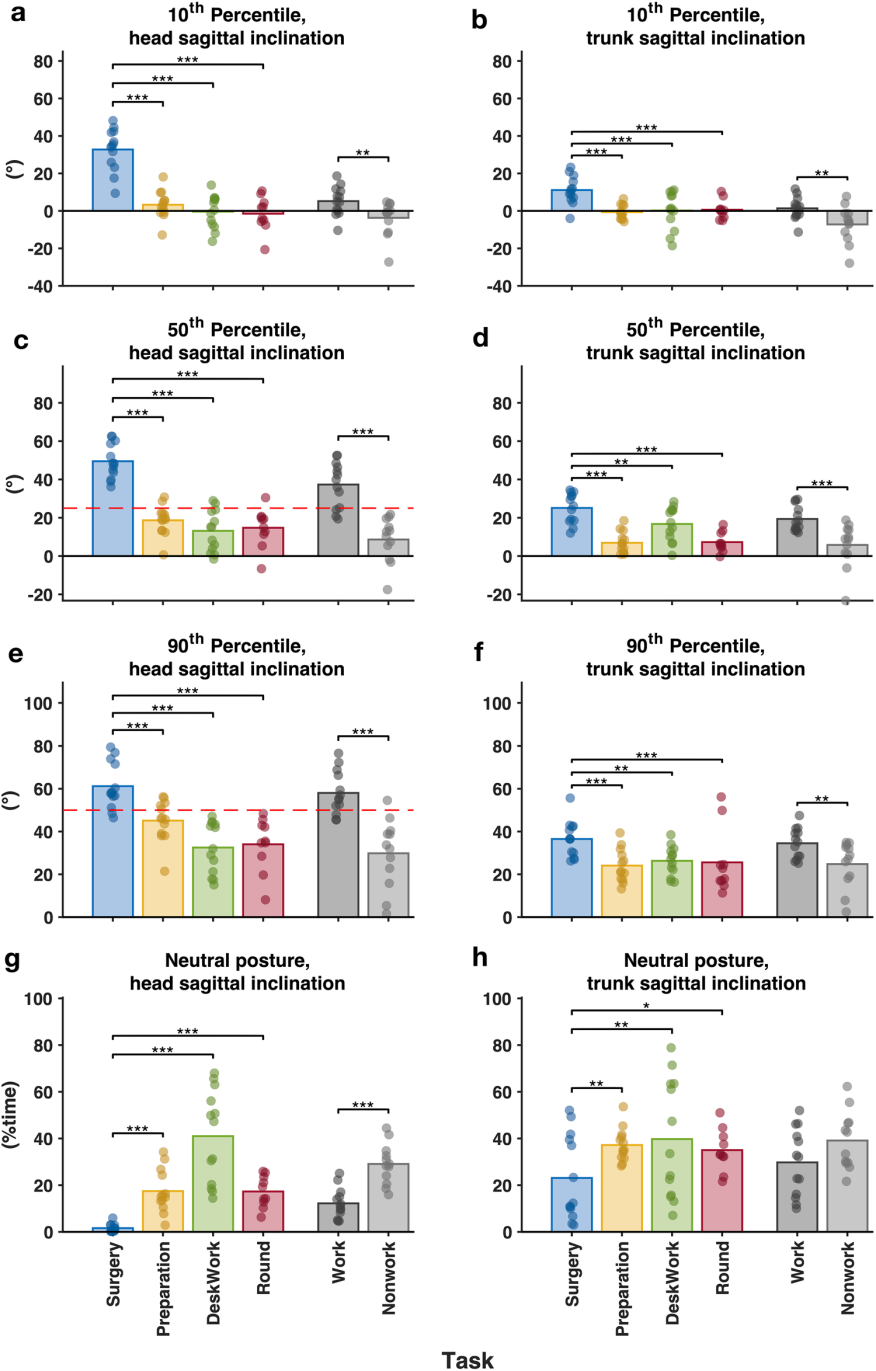
Comparisons of group means of postures between individual tasks and between work and nonwork activities. The bars represent the mean value of the group and the filled circles represent the individual value of the group. The red dashed line denotes the action level proposed by Arvidsson, Dahlqvist [30]. *p* values are denoted as * < 0.05; ** < 0.01; *** < 0.001 (Color figure online)

In Fig. 4, the postures and movements of the head and trunk are compared between surgery and nonsurgical tasks and between work tasks and nonwork activities.

Surgery was associated with a significantly larger head sagittal inclination angle than nonsurgical tasks at the 10th, 50th, and 90th percentiles. The 50th and 90th percentiles of the head sagittal inclination angle were significantly higher than the proposed action level (i.e., 25° and 50° for the 50th and 90th percentiles, respectively). The 50th percentile of the head sagittal inclination velocity during surgery was significantly lower than those during surgical preparation and rounds but significantly higher than that during desk work (see Table 5 in Appendix). The time proportion of neutral head postures during surgery was significantly lower than that during nonsurgical tasks. Overall, the 10th, 50th, and 90th percentiles of the head sagittal inclination angles during work tasks all exceeded those during nonwork activities. The 50th and 90th percentiles of the head sagittal inclination angles during work tasks exceeded the proposed action levels (i.e., 25° and 50° for the 50th and 90th percentiles). The work tasks also had a higher 50th-percentile head velocity and less head neutral posture time than nonwork activities (see Table 5 in Appendix).

For the trunk, the 10th, 50th and 90th percentiles of the trunk sagittal inclination angles during surgery were significantly higher than those during nonsurgical tasks. The proportion of time in a neutral trunk posture during surgery was significantly lower than during nonsurgical tasks. The 50th percentile of the trunk velocity during surgery was significantly higher than that during surgical preparation and rounds but significantly lower than that during desk work (see in Table 5 Appendix). All three percentiles of the trunk sagittal inclination angles and the 50th percentile of trunk velocity during work tasks were significantly higher than those during each of the nonwork activities. The proportion of time spent in a neutral trunk posture during work tasks was not significantly different from that spent during nonwork activities (see Table 5 in Appendix).

For the dominant arm, there were no significant differences between the 10th, 50th and 90th percentiles of the elevation angle during surgery and those during any of the three nonsurgical tasks. The 50th percentile of the elevation velocity of the dominant arm during surgery was significantly higher than those during surgical preparation and rounds but significantly lower than that during desk work. Both the 50th percentile and the 90th percentile of the arm elevation angle and the 50th percentile of the arm elevation velocity were below the proposed action levels for the corresponding measures.

### Part II: Modality comparison

The average surgical durations (mean ± SD) of the chief and assistant roles in RAS (chief: 134 ± 46 min, assistant: 158 ± 44 min) were significantly (*p* < 0.001, *p* < 0.001) longer than that of open neck surgeons (67 ± 43 min).

**Fig. 5:**
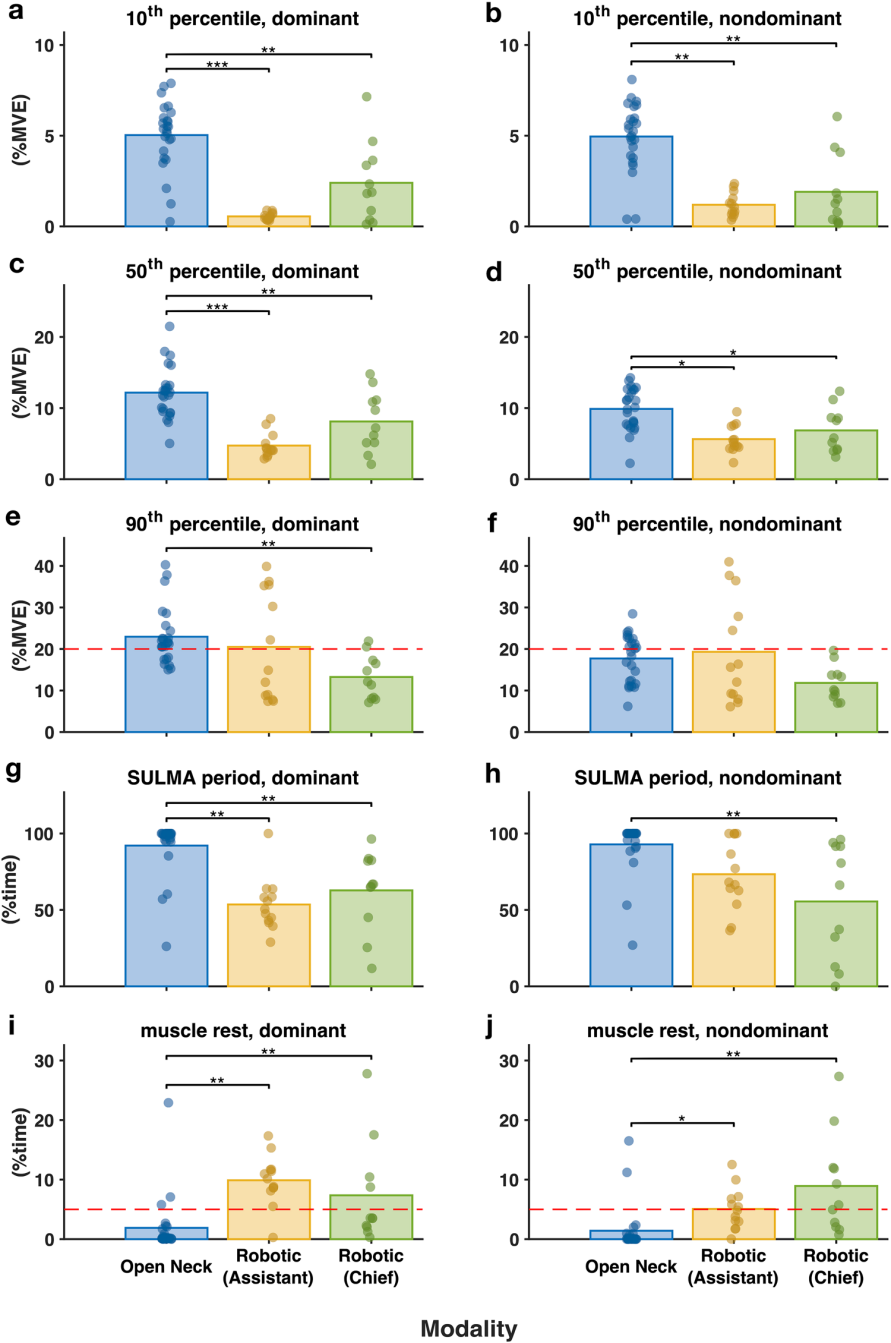
Muscle activity in the trapezius in open surgery and in the two roles in robotic surgery. The bars represent the mean value of the group and the filled circles represent the individual value of the group. The red dashed line denotes the action level proposed by Arvidsson, Dahlqvist [30]. *p* values are denoted as * < 0.05; ** < 0.01; *** < 0.001 (Color figure online)

In Fig. 5, the muscle activity of the trapezius was compared between surgeons performing open surgery and surgeons performing two different roles in RAS.

On the dominant side, open surgery had significantly higher static muscle activity levels (5.0%MVE) and median muscle activity levels (12.2%MVE) than the chief role (static: 2.4%MVE, median: 8.1%MVE) and assistant role (static: 0.5%MVE, median: 4.7%MVE) in RAS. The peak muscle activity level in open surgery (23.0%MVE) was also significantly higher than that for the chief role (13.2%MVE) in RAS, but not significantly different from that for the assistant role (20.5%MVE).

On the nondominant side, only the static muscle activity level and the median muscle activity level of the trapezius in open surgery were significantly higher than those for the two roles in RAS.

The peak trapezius muscle activity levels of both the dominant and nondominant sides in all three modality categories were not significantly higher than the proposed action level of 20%, with the *p* value for the dominant side in open surgery being above 0.05 and the p values for the dominant sides in both roles in RAS being above 0.3.

The SULMA time of the dominant trapezius in open surgery was 92%, which significantly exceeded that for both the chief role (63%) and the assistant role (54%) in RAS. Furthermore, the SULMA time of the dominant trapezius was significantly higher in open surgery (93%) than of the chief role in RAS (56%), but it was not significantly different from that of the assistant role in RAS. The muscle rest time of the trapezius in open surgery was significantly lower (1.4–1.9%) than that for both roles (5.1–9.9%) in RAS on both sides.

The muscle rest time of the trapezius in open surgery (dominant side: 1.9%, nondominant side: 1.4%) was below the proposed action level for muscle rest time (5%) [31], while the two roles in RAS both had rest times that were similar or higher than the action level (i.e., above 5%).

## Discussion

In comparison to nonsurgical tasks, surgery required significantly higher muscle activity and less muscle rest time in the trapezius, more awkward postures and less neutral posture time in the head and the trunk. The sagittal inclination angle of the head and the muscle activity of the trapezius during surgery were significantly higher than the recently proposed action levels by Arvidsson, Dahlqvist [31], while those during other nonsurgical tasks were not.

When compared to the chief role in RAS, open surgery required significantly higher levels of muscle activity, more SULMA time, and less muscle rest time in the trapezius. However, the differences in trapezius muscle activity between open surgery and the assistant role in RAS were less clear.

### Methodological considerations

This study collected measurements from 49 surgical cases and 13 workdays from 22 surgeons. The number of participants and the volume of measurements in this study were similar to those in several previous studies in which technical measurement instruments were deployed [11, 15, 16]. The participants also varied in terms of sex, age, and surgical experience, resembling the surgical population of the concerned specialty [47].

The current study examined one surgical specialty for each surgical modality; that is, open neck surgery was used for representing open surgery, and urological surgery was used for RAS. Szeto, Ho [17] used a mix of different specialties within each surgical modality. Using such a mix might have improved the generalizability of the results, but it would at the same time introduce more uncertainty. By using only one surgical specialty, the surgical cases in the current study were more homogeneous with lower variation compared to those expected with a multiple specialty study design.

All participants were followed during surgical workdays. The term “surgical workday” is not generally used in hospitals but is specific to this study. Since surgeons in this study worked in academic hospitals, they bore responsibilities for research and teaching in addition to surgery. Their workdays were scheduled such that tasks that were related to similar responsibilities and occurred in one location were arranged on the same day. A typical week for the participants in this study includes three to four surgical workdays, and the rest of the day(s) may vary week by week. The tasks performed in a surgical workday were common tasks that all surgeons around the world share, while the tasks performed on other workday(s) may vary in different hospitals. Therefore, only surgical workdays were included in this study.

The task-based approach of this study enabled decomposition and quantification of the workload of each component of a surgical workday. This approach may help to differentiate high-risk tasks from low-risk tasks, which may aid ergonomists, managers, engineers, designers and surgeons in identifying key issues of current surgical techniques and enable them to develop strategies for improving specific part(s) of surgeons’ workflow while keeping other functional and established parts of the workflow intact.

The assessments of physical workload in this study were based on technical measurements, that is, accelerometry and EMG, which generally produce precise and reliable measurements [41]. The workload of the shoulder was reflected from both the muscular perspective (trapezius activity) and the kinematic perspective (postures and movements), individually. An integration of both measurements might offer more insights, but as Merkus, Mathiassen [48] showed, the benefit gained from the composite metric of the two shoulder loads may be limited.

Posture and movement were only assessed via accelerometers. As demonstrated by several studies [40, 49, 50], accelerometers alone may produce biased results, especially when there are high angular velocities, while they are less prone to bias at lower velocities and for posture measurement. Given the relatively low velocities in the current study, the biasing of results from using accelerometers alone is limited, especially for posture.

The acquired data were not fully balanced between modalities and tasks. To overcome this issue, a mixed-effects model was adopted to test whether certain modalities or tasks were significantly different from the reference modality/task. Such a model has been used in several recent studies to overcome similar statistical issues [15, 51].

### Open surgery versus nonsurgical occupations

#### Workload in tasks

This study shows that surgeons bend their head more than 50° for half of the surgery time on average and surgery alone occupies more than half of a surgical workday, which exceeds the measured inclination angle among dentists performing patient treatment tasks (39°) [28] and dental hygienists performing various direct patient treatment tasks (21°–40°) [27]; the dental professionals, have a high prevalence of MSDs and high mechanical exposures [28, 52]. The 50th percentile of head inclination during surgery in this study is approximately twice as high as that during assembly work (20°–25°) [53], and it also substantially exceeds the proposed action level of 25° [31], which indicates an increased risk of MSDs in the neck/shoulder region.

The awkward posture of the head in surgery contrasts with the low median head sagittal inclination angle in nonsurgical tasks (13°–19°). The latter is similar to or less than that in other similar nonpatient treatment tasks, such as administration tasks (20°), handling parts and materials (24°), and disturbances (20°), among dentists [28]. These head inclination angles are similar to or lower than the corresponding proposed action level [31].

The angular velocities of the head, upper arms and trunk in the surgical task were significantly lower than those in nonsurgical tasks, which are also lower than those among assembly workers (14.1–30.4°/s) [53] and similar to those during patient treatment tasks among dentists (2.7–7.7°/s) [28] and dental hygienists (5.8–13.2°/s) [27].

Surgery was also associated with higher muscle activity levels, a greater proportion of SULMA time, and less muscle rest time in the trapezius than nonsurgical tasks.

These results collectively depict open surgery as a work task that requires a static and awkward work posture for the neck, exposing surgeons to a sustained load in the shoulders and spine.

Noticeably, the median arm elevation (21°–26°) in the surgery was lower or similar than that during other nonsurgical tasks and lower than that for dentists during patient treatment (28°–33°) [28] and for assembly workers during assembly work (27°–32°), and the arm elevation in the surgerywas also below the proposed action level (30°). These observations, which are in contrast to the high prevalence of MSDs among surgeons, suggest that the extent of arm elevation alone may not be an important factor for the development of MSDs in surgeons’ shoulders. The high precision demands and the large proportion of SULMA time might be important.

#### Open neck surgeons as an occupation

The work tasks measured in this study occupied most (82%) of the surgical day. During those work tasks, the time proportion of SULMA of the open neck surgeons was larger than that of those in other occupations, such as electricians (20–21%) and hairdressers (40–44%), on both sides [20]. The surgeons also had less trapezius muscular rest time (5–8%) than dental hygienists (8–9%) [27, 54], cashiers (10%, right side) [54] and hairdressers (7%, right side) [54] but more muscular rest time than CAD workers (4%, right side) [54], hospital cleaners (3%, right side) [54] and hotel cleaners (2%, right side) [54].

Regarding head postures, open neck surgeons had a median head inclination of 37° during work tasks on surgical workdays, which is similar to or higher than that of dentists (29°–39°) [28, 54] and dental hygienists (26°–27°) [27, 54]. This angle was also higher than that observed in those in other hand-intensive occupations, such as office/computer workers (22°), fish bone removers (18.5°), and cashiers (11°) [54], and other physically demanding occupations with a high frequency of disorders, such as hotel cleaners and hospital cleaners (26°–30°) [54].

Large proportions of SULMA time [55, 56], low muscle rest time in the trapezius [57], and high head/neck inclination [31] have been associated with an increased prevalence of neck pain and shoulder pain. The results from this study demonstrate the heavy physical loads in the neck of surgeons during open surgery and highlight the risks of developing MSDs.

### Workload of open surgery and robot-assisted surgery

#### Open surgery versus robot-assisted surgery

In this study, the bilateral levels of trapezius muscle activity in open surgery were slightly lower than those reported in open surgery by Szeto, Ho [17], who reported levels of 7.5/10%MVE (estimated) at the static level and 15/20%MVE at the median level. The difference could be due to the inclusion of a variety of types of open surgery in Szeto, Ho [17], especially abdominal surgeries that involve larger organs and larger surgical sites, while this study only included open neck surgery. The trapezius muscle activity levels in RAS were, in the current study, similar to those in RAS as reported by Dalager, Jensen [16].

Nevertheless, comparing to RAS, the open surgery in this study shows higher peak muscle activity level in the dominant trapezius (comparing to the chief role), less muscle rest time and more SULMA time on both sides of trapezius, and much lower head (49.5°) and trunk (25.1°) sagittal inclination than the neck flexion (4–13°) and trunk flexion (− 1° to 0) from a previous study [30]. Although head sagittal inclination cannot be directly compared with neck flexion, these large differences still indicate a higher postural neck load for open surgeons than for robotic surgeons.

The peak trapezius muscle activity of the open surgeon was also similar to the proposed action level of 20% [31], and the trapezius muscle rest time (1.4–1.9%) was significantly lower than the proposed action levels of 5% [31].

All these results indicate that, compared to open surgery, RAS generally demands less trapezius muscle activity, offers more time for muscle rest in the trapezius, and requires less awkward postures for the head, and consequentially the neck and spine.

High levels of trapezius muscle activity in combination with little muscle recovery time and a prolonged time spent with awkward head postures are associated with an increased risk of MSDs in the neck and shoulder [12, 13]. These muscular and postural loads indicate a higher MSD risk among open neck surgeons than among robotic surgeons. However, this inference is contrasted by a study in which surgeons performing minimally invasive surgery suffered significantly more than those performing open surgery [1]. One reason for these contradictory findings could be the collapse of RAS and laparoscopic surgery into one category, as Wells, Kjellman [9] has shown that robotic surgeons have fewer physical complaints than laparoscopic and open surgeons. This could also partially be due to the prolonged surgical procedure time in RAS compared to open surgery [58–60], since long surgical durations, with little muscular rest, are associated with higher MSD rates [61]. More studies are, therefore, needed to investigate the interactions of multiple risk factors and their impacts on the overall risks of MSDs.

#### Two roles in robot-assisted surgery

In comparison to the chief surgeons in RAS, the assistant surgeons appear to have less adverse working conditions—they had a larger variance in trapezius muscle activity, with lower static and median muscle activity levels and more muscle rest time. However, the assistant surgeons had higher peak muscle activity levels than the chief surgeons, and nearly half of the measured cases were above the proposed action level of 20%MVE. A similar difference was also shown by Yu, Dural [30], where the assistant surgeons had a higher neck flexion than the chief surgeons, while the chief surgeons had a lower range of motion in the neck, indicating that chief surgeons seem to have more constrained postures. The comparisons performed among modalities/roles indicate that both chief and assistant surgeons may benefit from RAS compared to conventional surgery, such as open surgery. However, since high postural loads and high peak muscle activity are evident physical risk factors for shoulder or neck disorders [12, 13], the health benefits for each role may differ. It is also unknown how much the workload is reduced and shared by robotic instruments when transitioning from open surgical procedures to robotic procedures and how much the workload was merely shifted from being part of the chief role to being part of the assistant role. An in-depth workload assessment is needed to identify these details.

### Surgery management and rationalization

The progress of rationalization in private and public services [26, 62] and the high demands of surgical tasks have resulted in increased challenges in surgery management. Rationalization, driven by increased demands for cost efficiency, pushes for an increase in VAW in production [22, 62, 63]. For surgical departments in hospitals, this is often realized as an increase in the efficiency of use of the operating room, which brings financial benefits to the hospital [23].

However, this increased proportion of VAW in operating rooms may lead to an increased proportion of surgical tasks in work hours for surgeons [23]. Consequently, as shown in this study, surgeons are then exposed to an increased time in sustained workload in the trapezius, increased time in awkward head/neck postures; hence, less time in VAWs, providing possibilities for recovery. Such work intensification has been reported in other industries, such as car disassembly [64]. The results of this study highlight the potential adverse health effects among surgeons following management strategies that prioritize efficiency in operating rooms.

A possible approach to reduce the negative health consequences following work intensification could be to accelerate a shift from open surgeries to minimally invasive surgeries; the latter have been consistently shown to place lower physical demands on surgeons [14, 15, 17]. However, such a shift in surgical modality could introduce other negative effects, such as increasing surgical duration [58–60], which reverses partial benefits brought by rationalization. The shift is also constrained by practical and medical reasons. Therefore, another possible approach could be to reduce the workload for certain tasks by introducing technical solutions such as the use of prismatic lenses [65–67] or increasing recovery time during surgeries, e.g., microbreaks [68], without modifying the surgical modality. The latter could be both timely and economically efficient and globally applicable in the foreseeable future.

This study showed that open surgery results in increased physical workloads compared to other nonsurgical work tasks. This was expressed as higher muscle activity levels of the trapezius, less muscle rest time, a higher proportion of time in sustained muscle activity, and more demanding postures for the head and trunk. The results of this study indicate that further rationalizations with a focus on increasing the proportion of time in surgery (VAW) will increase the physical workload in surgery, which may further increase the risk of work-related MSDs among surgeons.

This study also showed that open surgery induces higher physical workloads than RAS in terms of trapezius muscle activity. The trapezius activity in open surgery is also high in comparison to that observed in many other occupations, and the trapezius muscle rest time is significantly shorter than the corresponding proposed action level. This indicates an elevated risk for neck-shoulder pain for open surgeons. When comparing the workload between the chief and assistant roles in RAS, the assistant role induced a higher peak load, while the chief role induced higher static loads with the least muscular rest time. Hence, they both had unique advantages and disadvantages, which adds to the complexity of the load patterns.

Shifting from open surgery to RAS may, therefore, lower trapezius muscle activity for chief surgeons, but it is not yet adequate to minimize surgeons’ risks for developing MSDs overall. As open surgeries will still be the major surgical modality in the foreseeable future, interventions for minimizing the MSD risks for open surgery, such as improved technical devices (such as prismatic loupes), work organization changes (such as improved scheduling) and microbreaks, should be encouraged.

### Sex-inclusive biomedical and clinical research

The study has a sex-inclusive study base constituting on average 30% women with certain subgroup of 40% women. 30% women in the surgeon population can be considered as representative [69].

## Appendix

See Table 5.

**Table 5:** Comparisons of postural and movement workload of open neck surgeons among tasks

	Task								Work		Nonwork	
	Surgery		Preparation		Desk work		Rounds					
	Mean	SD	Mean	SD	Mean	SD	Mean	SD	Mean	SD	Mean	SD
*Upper arm, Dominant*												
Elevation												
Percentile (°)												
10th	11.2	3.9	12.7	5.8	15.3**	5.1	11.0	4.4	11.8^a^	4.2	15.3	6.3
50th	26.2	7.4	23.8	7.0	25.3	7.9	22.7	6.0	25.1^a^	6.7	29.6	8.7
90th	46.2	11.2	53.8	11.2	41.0	10.6	45.2	14.6	45.9	8.6	46.4	12.8
%Time at neutral posture	18.9	10.0	16.8	8.4	24.3	21.4	17.3	9.9	18.8^aa^	9.9	10.4	7.3
Movement												
Percentile (°/s)												
50th	5.8	1.3	14.3***	6.0	2.9***	1.5	10.9**	4.5	6.1	1.3	7.0	2.4
%Time at low velocity	46.6	5.9	31.1***	12.6	63.2***	8.1	36.1**	9.5	45.8	4.7	43.6	8.1
*Upper arm, non-Dominant*												
Elevation												
Percentile (°)												
10th	8.7	2.3	12.6**	3.6	11.5*	3.2	10.5	3.5	9.1^aa^	1.9	13.3	4.3
50th	21.1	4.2	23.8	6.1	22.7	6.3	21.8	6.3	21.3^aaa^	3.3	34.3	17.0
90th	38.8	8.0	54.2***	11.9	43.2	8.7	45.8	11.6	41.6^a^	6.8	51.0	14.8
%Time at neutral posture	28.3	7.5	17.3**	8.7	30.0	14.9	17.7**	8.0	26.2^aa^	6.3	15.5	12.1
Movement												
Percentile (°/s)												
50th	4.4	1.0	12.9***	5.0	2.9***	1.2	9.7***	3.2	5.0	1.2	6.0	2.4
%Time at low velocity	54.3	7.1	32.3***	12.1	64.0***	8.5	37.8***	7.2	50.5	5.5	47.5	8.8
*Head*												
Sagittal inclination												
Percentile (°)												
1st	8.9	11.5	-8.7***	8.8	-10.9***	9.7	-11.5***	9.4	-8.8	8.0	-13.3	9.0
10th	32.7	11.2	3.3***	7.4	-0.4***	8.8	-1.5***	9.2	5.2^aa^	7.8	-3.7	9.3
50th	49.5	9.1	18.7***	7.7	13.1***	10.7	14.8***	10.1	37.3^aaa^	11.8	8.6	11.5
90th	61.2	10.8	45.1***	9.5	32.5***	12.0	34.0***	12.5	58.0^aaa^	10.1	29.8	16.0
%Time at neutral posture	1.6	1.7	17.4***	9.2	41.0***	19.7	17.3***	6.7	12.2^aaa^	6.4	29.1	8.7
Movement												
Percentile (°/s)												
50th	4.8	1.0	11.4***	3.2	3.9*	1.3	12.8***	2.5	5.7^aaa^	1.1	8.4	1.7
%Time at low velocity	52.2	6.6	30.6***	9.0	58.2*	8.4	29.4***	4.3	47.2^aaa^	5.0	37.9	5.4
*Trunk*												
Sagittal inclination												
Percentile (°)												
1st	1.1	6.6	-6.7***	3.2	-9.2***	8.5	-3.6*	4.7	-8.0^a^	6.3	-17.9	11.0
10th	11.1	7.4	-0.7***	3.7	0.2***	9.9	0.6***	5.1	1.4^aa^	6.0	-7.2	9.8
50th	25.1	7.9	6.9***	5.6	16.7**	8.9	7.3***	5.1	19.4^aaa^	6.5	5.8	11.6
90th	36.5	8.4	24.1***	7.7	26.3**	6.9	25.6***	15.1	34.5^aa^	7.1	24.8	10.8
%Time at neutral posture	23.1	18.2	37.2**	7.1	39.7**	25.3	35.0*	8.9	29.7	14.4	39.1	12.4
Movement												
Percentile (°/s)												
50th	3.0	0.6	6.9***	2.1	2.3**	0.8	7.4***	1.9	3.4^aaa^	0.7	5.1	1.5
%Time at low velocity	66.8	6.0	42.8***	10.2	70.8*	7.5	41.5***	6.2	60.7^aaa^	5.2	50.3	7.3

*, ** and *** are levels of p-values in comparison between a nonsurgical task and surgery. **p* < 0.05, ***p* < 0.01, ****p* < 0.001

^a^, ^aa^, and ^aaa^ are levels of p-values in comparison between work and nonwork. ^a^*p* < 0.05, ^aa^*p* < 0.01, ^aaa^*p* < 0.001
